# Permissive hypotension/hypotensive resuscitation and restricted/controlled resuscitation in patients with severe trauma

**DOI:** 10.1186/s40560-016-0202-z

**Published:** 2017-01-20

**Authors:** Daisuke Kudo, Yoshitaro Yoshida, Shigeki Kushimoto

**Affiliations:** 1 Division of Emergency and Critical Care Medicine, Tohoku University Graduate School of Medicine, 2-1 Seiryo-machi, Aoba-ku, Sendai, 980-8574 Japan; 2 Department of Emergency and Critical Care Medicine, Tohoku University Hospital, 1-1 Seiryo-machi, Aoba-ku, Sendai, 980-8574 Japan

**Keywords:** Controlled resuscitation, Delayed resuscitation, Hypotensive resuscitation, Permissive hypotension, Restricted resuscitation, Shock, Trauma, Trauma-induced coagulopathy

## Abstract

Achieving a balance between organ perfusion and hemostasis is critical for optimal fluid resuscitation in patients with severe trauma. The concept of “permissive hypotension” refers to managing trauma patients by restricting the amount of resuscitation fluid and maintaining blood pressure in the lower than normal range if there is continuing bleeding during the acute period of injury. This treatment approach may avoid the adverse effects of early, high-dose fluid resuscitation, such as dilutional coagulopathy and acceleration of hemorrhage, but does carry the potential risk of tissue hypoperfusion. Current clinical guidelines recommend the use of permissive hypotension and controlled resuscitation. However, it is not mentioned which subjects would receive most benefit from this approach, when considering factors such as age, injury mechanism, setting, or the presence or absence of hypotension. Recently, two randomized clinical trials examined the efficacy of titrating blood pressure in younger patients with shock secondary to either penetrating or blunt injury; in both trials, overall mortality was not improved. Another two major clinical trials suggest that controlled resuscitation may be safe in patients with blunt injury in the pre-hospital setting and possibly lead to improved outcomes, especially in patients with pre-hospital hypotension. Some animal studies suggest that hypotensive resuscitation may improve outcomes in subjects with penetrating injury where bleeding occurs from only one site. On the other hand, hypotensive resuscitation in blunt trauma may worsen outcomes due to tissue hypoperfusion. The influence of these approaches on coagulation has not been sufficiently examined, even in animal studies. The effectiveness of permissive hypotension/hypotensive resuscitation and restricted/controlled resuscitation is still inconclusive, even when examining systematic reviews and meta-analyses. Further investigation is needed to elucidate the effectiveness of these approaches, so as to develop improved treatment strategies which take into account coagulopathy in the pathophysiology of trauma.

## Background

### General concept of permissive hypotension and damage-control resuscitation

Traditionally, the concept of “early and aggressive” fluid administration has been applied to patients with severe trauma, in order to restore circulating blood volume and maintain tissue perfusion. However, this treatment approach may increase hydrostatic pressure in injured vessels, dislodge hemostatic blood clots [1, 2], induce dilutional coagulopathy [3, 4], and result in hypothermia [5]. The concept of “permissive hypotension” refers to managing trauma patients by restricting the amount of fluid resuscitation administered while maintaining blood pressure in the lower than normal range if there is still active bleeding during the acute period of injury [6, 7]. Although this treatment approach may avoid the adverse effects of early and high-dose fluid resuscitation, it carries the potential risk of tissue hypoperfusion.

**Fig. 1:**
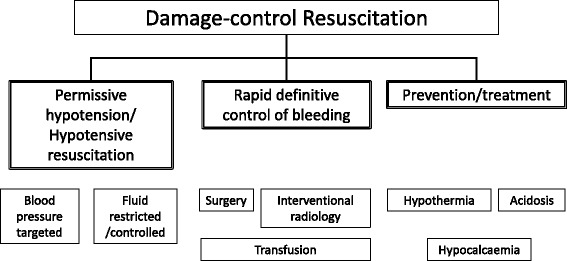
Main principles of damage-control resuscitation

“Permissive hypotension” is included in the overarching concept of “damage-control resuscitation.” The concept of damage-control resuscitation has been developed with the aim of providing optimal fluid resuscitation and transfusion to patients with hemorrhagic shock secondary to severe trauma [8–10]. The main principles are permissive hypotension/hypotensive resuscitation, rapid and definitive/surgical control of bleeding, and the prevention/treatment of hypothermia, acidosis, and hypocalcemia (Fig. 1). The goal of damage-control resuscitation is to minimize iatrogenic resuscitation injury, to prevent worsening of initial traumatic shock, and to obtain definitive hemostasis.

Attention should be brought to the fact that there are several similar-sounding terms included in the concept, such as “permissive hypotension/hypotensive resuscitation,” “restricted/controlled resuscitation,” and “delayed resuscitation” (Table 1). “Permissive hypotension/hypotensive resuscitation” implies the titration and control of blood pressure. “Restricted/controlled” resuscitation refers to the volume of fluid administered. “Early” or “delayed” resuscitation indicates the timing of resuscitation. In most studies, “early” implies initiating fluid resuscitation in the pre-hospital setting, while “delayed” is taken to mean starting fluid resuscitation after admission to hospital.

**Table 1:** Interventions to patients in each type of resuscitation strategy

Type of resuscitation strategy	Interventions to patients	Major clinical trials focusing on the concepts
Permissive hypotension, Hypotensive resuscitation	To titrate and control the blood pressure less than normal range	Dutton et al. 2002, Morrison et al. 2011
Restricted resuscitation, Controlled resuscitation	To limit the volume of fluid to be administered	Brown et al. 2013, Schreiber et al. 2015
Delayed resuscitation	To restrict the fluid resuscitation until admission to the hospital (Early resuscitation is opposite term that means to initiate fluid resuscitation from pre-hospital setting)	Bickel et al. 1994, Sampalis et al. 1997, Turner et al. 2000

Neither permissive hypotension/hypotensive resuscitation nor restricted/controlled resuscitation may be indicated in patients with traumatic brain injury (TBI) and/or spinal injury [11]. This is because resuscitation to maintain adequate perfusion is essential to ensure tissue oxygenation of the injured central nervous system and avoid secondary injury [8, 12, 13]. Most studies of permissive hypotension have excluded subjects with TBI and spinal injury, in both animal and clinical investigations. The current review will not consider TBI or spinal injury.

### History of permissive hypotension

Cannon et al. first described the use of permissive hypotension in patients with severe trauma in 1918 [14]. It has been shown that fluid resuscitation before achievement of hemostasis in injured patients may be harmful. It was originally supposed that target systolic blood pressure (SBP) prior to hemostasis should be maintained between 70 and 80 mmHg. However, based on animal studies in the 1950s and 1960s, major textbooks recommended early and aggressive resuscitation [15–17]. However, other studies from the 1960s had demonstrated that aggressive fluid resuscitation may in fact be detrimental in subjects with uncontrolled hemorrhage [18–22].

Bickel et al. conducted a prospective trial and reported that delaying aggressive fluid resuscitation until operative intervention improves outcomes in hypotensive patients with penetrating torso injury [23]. This prospective, controlled trial included 598 adults with penetrating torso injuries who presented with a pre-hospital SBP <90 mmHg. They compared delayed fluid resuscitation (started in the operating room) with immediate fluid resuscitation (initiated by paramedics in pre-hospital settings). The survival rate at discharge from the hospital was higher in patients who received delayed fluid resuscitation compared to those who received immediate fluid resuscitation. This study strongly suggested the effectiveness of restricted and delayed fluid resuscitation in patients with severe penetrating trauma, although it did not evaluate the efficacy of titrating blood pressure control.

Another two trials focused on the timing and administration of fluid started in pre-hospital settings or in-hospital in patients with blunt trauma including traumatic brain injury [24, 25]. Of the two trials, one observational study showed that the use of on-site intravenous fluid replacement was associated with an increased risk of mortality [24]. In another randomized, controlled study, no significant difference in survival was found, but this study was limited by a high protocol violation rate [25].

#### Clinical evidence and experimental findings of permissive hypotension

Several researchers have reported the effectiveness of permissive hypotension in clinical and experimental studies. In those studies, target values of blood pressure were various, and systolic or mean arterial pressure was defined as target blood pressure.

### Clinical trials for targeting and controlling blood pressure

Dutton et al. compared targeting of blood pressure values within normal ranges to below normal ranges in patients with severe trauma for the first time [26] (Table 2). Patients were eligible for inclusion if they had evidence of ongoing hemorrhage and had a SBP <90 mmHg recorded at least once within the first hour after injury. Eligible patients were randomized to two groups: fluid (crystalloid and blood products) administration titrated to either a conventional SBP >100 mmHg or a low SBP of 70 mmHg until definitive hemostasis was achieved. The survival rate did not differ in either group (each being 92.7%). This randomized controlled trial (RCT) thus showed that titration of initial fluid therapy to maintain a lower than normal SBP during active hemorrhage did not affect mortality. In this study, almost half of the subjects were injured by blunt trauma (49%) and the origin of hemorrhage was not only from chest and abdominal trauma but also included various other sites such ﻿as the retroperitoneum and lower limbs. Patients with central nervous system injury were excluded. The mean age of the subjects was 31, after excluding patients older than 55. Patients with pre-existing diabetes mellitus or coronary artery disease were also excluded. Based on the limitations of the study, the authors recommended that further investigation in this area should focus on specific patient populations most likely to benefit from deliberate hypotensive resuscitation. Coagulation was also not evaluated in this study.

**Table 2:** Summary of the clinical trials for permissive hypotension/hypotensive resuscitation and restricted/controlled resuscitation

Type of resuscitation strategy	Authors	Study design	Patients	Interventions	Control	Primary outcome	Secondary outcome/sub-analysis	Outcome for coagulations	Major limitations
Permissive hypotension, Hypotensive resuscitation	Dutton et al. 2002	RCT	With traumatic injury, ongoing hemorrhage, and had a SBP <90 mmHg	Fluid administration titrated to SBP of 70 mmHg	Fluid administration titrated to SBP of 100 mmHg	The overall survival rate did not differ	N.A.	N.A.	The mean age was 31, excluding patients older than 55 and medical history of diabetes or coronary artery disease
	Morrison et al. 2011	RCT	Undergoing emergency laparotomy or thoracotomy for trauma, who had SBP ≤90 mmHg	Fluid administration titrated to MAP of 50 mmHg	Fluid administration titrated to MAP of 65 mmHg	30-day mortality did not differ	Mortality in the early postoperative period was decreased, and received blood products were fewer	Immediate postoperative coagulopathy was less	Patients older than 45 years were excluded. 93% were penetrating injury
Restricted resuscitation, Controlled resuscitation	Brown et al. 2013	Post hoc analysis	With blunt injury, out-of-hospital SBP ≤90 mmHg, and ISS >15	Pre-hospital crystalloid resuscitation, ≤500 ml	Pre-hospital crystalloid resuscitation, >500 ml	30-day in-hospital mortality did not differ	Without pre-hospital hypotension, control group was associated with an increased risk of mortality	Without pre-hospital hypotension, control group was associated with an increased risk of acute coagulopathy	Post hoc analysis, only blunt trauma
	Schreiber et al. 2015	RCT	With blunt or penetrating injury and out-of-hospital SBP ≤90 mmHg	250 mL of fluid as an initial bolus, additional fluid to maintain an SBP of 70 mmHg	2 L of fluid as an initial bolus, additional fluid to maintain an SBP of 110 mmHg	The mean crystalloid volume administered was less	24-h mortality was decreased in patients with blunt trauma	Coagulation values at the emergency department did not differ	Feasibility and pilot study

Patients with traumatic brain injury were excluded in all of four trials

*RCT* randomized controlled trial, *SBP* systolic blood pressure, *N.A*. not applicable, *MAP* mean blood pressure, *ISS* injury severity score

Morrison et al. conducted a RCT determining the efficacy of hypotensive resuscitation [27] (Table 2). Target blood pressure was measured as mean arterial blood pressure (MAP). Patients undergoing emergency laparotomy and thoracotomy for blunt and penetrating trauma, who had had at least one in-hospital documented SBP ≤90 mmHg, were included in the study. Randomization occurred on arrival to the operating room, and the patients were treated as per standard of care. Crystalloid, colloid, and blood products were administered in fluid resuscitation. Patients assigned to receive a lower, 50-mmHg MAP target (defined as the LMAP group) received fewer blood products during intraoperative resuscitation than those assigned to receive a higher, 65-mmHg MAP target (defined as the HMAP group). The primary outcome was 30-day mortality and did not differ between the groups, although mortality in the early postoperative period was decreased in the LMAP group. Patients in the LMAP group were also less likely to develop immediate postoperative coagulopathy, as evaluated by partial thromboplastin time (PTT), prothrombin time (PT), and international normalized ratio (INR). This study showed that hypotensive resuscitation was a safe strategy in trauma patients, reduced the total amount of fluid and blood products used, and was associated with decreased postoperative coagulopathy. However, overall 30-day mortality was not improved in the LMAP group. This study had some limitations: standard treatment, including fluid resuscitation in the pre-hospital setting and emergency department, was performed in both groups. Therefore, fluid resuscitation received prior to arrival in the operating room may have influenced the results. The study also excluded patients older than 45 years, as well as any patients with potential traumatic brain injury. Of the patients studied, 93% were injured by penetrating trauma (gunshot wounds were the cause in 72.2% of cases).

### Animal studies in blood pressure titration

Some animal research has been undertaken in order to provide answers to the clinical questions raised about effectiveness of permissive hypotension/hypotensive resuscitation and investigate the results of clinical trials, as well as to examine the pathophysiological mechanisms and hemodynamics associated with hypotensive resuscitation. Sondeen et al. have shown that there was a reproducible pressure at which rebleeding occurred in a porcine model for penetrating injury [28]. Bleeding was induced by creating a hole in the aorta with a 1.5–2.8-mm skin biopsy punch. This study suggested that rebleeding might occur when blood pressure increases over a specific threshold value and that an increase in blood pressure above this value might result in the dislodging of any clots that had been formed at the site of injury.

Li et al. have however simultaneously shown the effectiveness of permissive hypotension in a rat model for splenic injury, induced by transection of the splenic parenchyma and one of the branches of the splenic artery [29]. One hundred thirty kilodaltons of hydroxyethyl starch and lactated Ringer’s solution (1:2) was administered in fluid resuscitation. The amount of bleeding and mortality was decreased in rats targeted to a mean arterial blood pressure of 50 mmHg compared with those in which the target was 80 mmHg. Coagulation values (thrombin time, INR, fibrinogen, PTT, platelet count, and aggregation) were similar in both groups.

Schmidt et al. investigated regional organ perfusion in the acute phase by investigating uncontrolled hemorrhage in a rat model for penetrating vascular injury and simulating pre-hospital times in urban trauma [30]. Bleeding was induced by a single puncture injury to the infra-renal aorta with a 25G needle. Lactated Ringer’s solution was administered in fluid resuscitation. In the permissive hypotension group, blood pressure was targeted to 60% of baseline MAP, compared with the normotensive (NBP) resuscitation group. Perfusion of any organ including the brain, heart, lung, kidney, liver, and bowel was similar in both groups. Cardiac output and lactate levels did not differ in either group. Intra-abdominal blood loss was higher in the NBP group. This study revealed that hypotensive resuscitation was able to maintain equivalent organ perfusion to normotensive resuscitation and caused less intra-abdominal bleeding than normotensive resuscitation.

However, Garner et al. have revealed an inverse of these results in a porcine model for primary blast injury [31]. In their study, all pigs sustained a controlled hemorrhage of 30% blood volume. 0.9% saline was administered in fluid resuscitation. The mortality in pigs targeted to an SBP of 80 mmHg was higher than those who were targeted to an SBP of 110 mmHg. A profound metabolic acidosis was also observed in the low target blood pressure group. This study has suggested that prolonged hypotensive resuscitation may negatively influence survival after primary blast injury.

The evidence from these animal studies suggests that hypotensive resuscitation may lead to improved outcomes in subjects with penetrating injury where bleeding occurs from only one site; on the other hand, it is possible that hypotensive resuscitation may worsen outcomes in blunt injury owing to tissue hypoperfusion. The influence of this technique on the coagulation system has not been sufficiently examined.

### Clinical trials examining restricted/controlled resuscitation

In the last several years, some retrospective analyses have demonstrated that aggressive resuscitation, often initiated in the pre-hospital setting, may be detrimental to trauma patients [32–35]. Recently, two studies have investigated the efficacy of restricted or controlled resuscitation in patients with trauma in the pre-hospital setting.

Brown et al. examined the effect of high vs low volume crystalloid resuscitation in the pre-hospital setting [36] (Table 2). This study is a post hoc analysis of a multicenter, prospective, cohort study of adults who sustained blunt trauma with hemorrhagic shock, designed to elucidate the genomic and proteomic responses following injury [37] using propensity-adjusted regression analysis. Adult patients with blunt injury transported from the scene and those with injuries of ISS >15 were included in the study. Subjects were divided into “HIGH” (>500 ml) and “LOW” (≤500 ml) pre-hospital crystalloid resuscitation groups. In subjects without pre-hospital hypotension (<90 mmHg), HIGH crystalloid was associated with an increased risk of mortality and acute coagulopathy (INR >1.5), but this was not seen in subjects with pre-hospital hypotension. The authors suggested that pre-hospital resuscitation should be goal-directed, based on the presence or absence of pre-hospital hypotension in severely injured blunt trauma patients.

Schreiber et al. performed a randomized pilot trial to compare the effect of controlled resuscitation (CR) with standard resuscitation (SR) in the pre-hospital scene [38] (Table 2). The mean (standard deviation) crystalloid volume administered during the pre-hospital period was 1.0 (1.5) L in the CR group and 2.0 (1.4) L in the SR group. Twenty-four-hour mortality was decreased in the CR group in patients with blunt trauma, whereas this was not seen in patients with penetrating injury. Overall in-hospital mortality did not differ between groups. Coagulation values (PTT, INR, and platelet counts) on admission to the emergency department did not differ between the groups. This pilot study suggested that a controlled resuscitation strategy can be successfully and safely implemented in the pre-hospital setting.

These two trials suggest that controlled resuscitation in patients with blunt injury in the pre-hospital setting may be safe and possibly improves outcomes, especially in patients with pre-hospital hypotension. Whether controlled resuscitation strategies improve survival and coagulopathy is yet to be determined in a large-scale phase III trial.

### Systematic review and meta-analysis

The Cochrane Library has published a systematic review examining the effect of fluid management on mortality and coagulation times in hemorrhagic hypovolemia, comparing both early vs delayed resuscitation and larger vs smaller volumes of fluid [39]. Only two trials examining the timing of administration [23, 25] and three trials investigating the amount of fluid volume [26, 40, 41] were included. The authors did not combine the results quantitatively, and meta-analysis was not performed, because the interventions and patient populations were so diverse.

A further meta-analysis [42] assessed three prospective studies [23, 26, 27] and seven retrospective observational studies [24, 43–47]. The quantitative synthesis indicated that initial liberal fluid resuscitation strategies might be associated with higher mortality when compared to restricted fluid strategies, both in RCTs and observational studies in trauma patients. However, the authors cautioned that the available studies are subject to a high risk of selection bias and clinical heterogeneity. Therefore, the effectiveness of permissive hypotension/hypotensive resuscitation and restricted/controlled resuscitation is still inconclusive, even when considering systematic reviews and meta-analyses.

### Guidelines

For patients with major trauma, defined as having an Injury Severity Score of more than or equal to 16, the American College of Surgeons’ Advanced Trauma Life Support (ATLS) guidelines currently advocate “balanced” resuscitation with an initial 1–2 L of crystalloids before definitive/surgical control of bleeding [48]. The mechanism of injury, whether penetrating or blunt, and injury site is not specifically described. Permissive hypotension and restricted fluid resuscitation strategies are stated in the fourth edition of the European guideline on management of major bleeding and coagulopathy following trauma [6]: “We recommend a target systolic blood pressure of 80–90 mmHg until major bleeding has been stopped in the initial phase following trauma without brain injury. (Grade 1C)”; “In patients with severe TBI (GCS ≤8), we recommend that a mean arterial pressure ≥80 mmHg be maintained. (Grade 1C)”; and “We recommend use of a restricted volume replacement strategy to achieve target blood pressure until bleeding can be controlled. (Grade 1B).” Restricted fluid resuscitation to achieve specific target blood pressure is highly recommended in this guideline, although injury mechanism and age are not specifically stated. The guidelines also recommend that serum lactate and/or base deficit measurements be used as sensitive tests to estimate and monitor the extent of bleeding and shock (grade 1B) [6]. However, there has been no report that investigates the lactate-oriented fluid management in trauma patients.

### Problems and areas of uncertainty

There are currently several problems and areas of uncertainty. Firstly, there are similar-sounding terms described in the introduction that are easily confounded, and while they apply to similar concepts, they differ slightly in their approach and targets. Understanding the definitions of these terms is vitally important. Furthermore, unification, standardization, and rearrangement of these terms by the authorities are also needed to ensure they are easily understood.

It remains to be seen whether it is actually possible to control blood pressure effectively in patients with active bleeding, where the physiological response attempts to maintain perfusion volume by secreting catecholamines that increase the cardiac output and induce vasoconstriction. In two clinical trials [26, 27] and three animal experiments [29–31], the actual blood pressure was indeed much higher than the target blood pressure. This suggests, at least, that target blood pressure is not an endpoint in itself.

The important factor may be the volume of fluid administered as a result of targeting low blood pressure. The strategy of restricted/controlled resuscitation may be more appropriate than that of permissive hypotension/hypotensive resuscitation. In addition, appropriate interventions, devices, and biomarkers for assessing tissue perfusion and ischemic risk do not currently exist, and this presents a difficulty in balancing tissue perfusion with the risk of adverse events in fluid resuscitation. The development of these devices and biomarkers is desired. Moreover, attention should be also paid to factors such as age, mechanism of injury (penetrating or blunt), and severity of injury, with or without the presence hypotension and controlled bleeding, as well as the setting in which trauma is attended to (pre-hospital, emergency department, or operating room). Permissive hypotension/hypotensive resuscitation may need to be used carefully in elderly patients and patients with chronic hypertension [49].

## Conclusions

At present, the effectiveness of permissive hypotension/hypotensive resuscitation and restricted/controlled resuscitation has not yet been completely proven. With regard to the resuscitating trauma patients in each study, consideration should be given to age, mechanism and severity of injury, presence or absence of shock, and whether treatment occurred at a pre-hospital or in-hospital setting. It needs to be elucidated what subsets of patients will be most likely to benefit from permissive hypotension/hypotensive resuscitation and restricted/controlled resuscitation. The impact of fluid resuscitation on coagulation also needs to be clarified. Achieving a balance between organ perfusion and hemostasis is critical when instituting fluid resuscitation in patients with severe trauma. It is hoped that further research will uncover optimal fluid resuscitation strategies for trauma patients.

## Abbreviations

INR: International normalized ratio
MAP: Mean arterial blood pressure
PTT: Partial thromboplastin time
RCT: Randomized control study
SBP: Systolic blood pressure
TBI: Traumatic brain injury
